# Optimizing Multivariable Logistic Regression for Identifying Perioperative Risk Factors for Deep Brain Stimulator Explantation: A Pilot Study

**DOI:** 10.3390/clinpract15070132

**Published:** 2025-07-17

**Authors:** Peyton J. Murin, Anagha S. Prabhune, Yuri Chaves Martins

**Affiliations:** 1 Department of Neurology, Saint Louis University School of Medicine, St. Louis, MO 63104, USA; peyton.murin@slucare.ssmhealth.com (P.J.M.); anagha.prabhune@slucare.ssmhealth.com (A.S.P.); 2 Department of Anesthesiology, Saint Louis University School of Medicine, St. Louis, MO 63110, USA

**Keywords:** deep brain stimulation, statistical modeling, movement disorders, surgical complications

## Abstract

**Background/Objectives**: Deep brain stimulation (DBS) is an effective surgical treatment for Parkinson’s Disease (PD) and other movement disorders. Despite its benefits, DBS explantation occurs in 5.6% of cases, with costs exceeding USD 22,000 per implant. Traditional statistical methods have struggled to identify reliable risk factors for explantation. We hypothesized that supervised machine learning would more effectively capture complex interactions among perioperative factors, enabling the identification of novel risk factors. **Methods**: The Medical Informatics Operating Room Vitals and Events Repository was queried for patients with DBS, adequate clinical data, and at least two years of follow-up (*n* = 38). Fisher’s exact test assessed demographic and medical history variables. Data were analyzed using Anaconda Version 2.3.1. with pandas, numpy, sklearn, sklearn-extra, matplotlin. pyplot, and seaborn. Recursive feature elimination with cross-validation (RFECV) optimized factor selection was used. A multivariate logistic regression model was trained and evaluated using precision, recall, F1-score, and area under the curve (AUC). **Results**: Fisher’s exact test identified chronic pain (*p* = 0.0108) and tobacco use (*p* = 0.0026) as risk factors. RFECV selected 24 optimal features. The logistic regression model demonstrated strong performance (precision: 0.89, recall: 0.86, F1-score: 0.86, AUC: 1.0). Significant risk factors included tobacco use (OR: 3.64; CI: 3.60–3.68), primary PD (OR: 2.01; CI: 1.99–2.02), ASA score (OR: 1.91; CI: 1.90–1.92), chronic pain (OR: 1.82; CI: 1.80–1.85), and diabetes (OR: 1.63; CI: 1.62–1.65). **Conclusions**: Our study suggests that supervised machine learning can identify risk factors for early DBS explantation. Larger studies are needed to validate our findings.

## 1. Introduction

Deep brain stimulation was first shown to be effective as a therapeutic option in essential tremor and Parkinson’s Disease (PD) in 1987 [1]. Since its initial use, the indications for DBS have expanded to include generalized dystonia, tremors, chronic pain, psychiatric disorders, and early PD, leading to a growing number of procedures [2,3,4,5,6]. Advancements in DBS technology, such as enhanced battery life, adjustable stimulation parameters, and directional stimulation, have further improved its efficacy [7]. In addition, ongoing developments, including closed-loop adaptive stimulation, continue to refine and expand its clinical applications [7].

However, although DBS is generally considered a safe procedure [8], complications can still occur. Previous studies found a mortality rate of 0.2% and a permanent morbidity rate of 0.6%, with 12.2% of patients having hardware complications [2]. Adverse effects of DBS include cognitive decline [9], ineffective stimulation/device dysfunction [10], infection [10], delirium [11], and hemorrhage [11], amongst others. Although explantation is relatively uncommon, it poses a substantial financial burden on the healthcare system, with each case estimated to cost over USD 22,000 [8]. Given that the combined rate of revision or explantation approaches 4.4% [12], there is a clear need to better identify patients at risk for these outcomes. While the previous literature has identified risk factors for DBS complications, including advanced age, male sex, and comorbidities such as dementia, hypertension, and coagulopathy, only elevated body mass index (BMI) has been demonstrated to be a risk factor for explantation itself [13,14]. As such, there remains a need to precisely determine which patients are at high risk for early explantation. To address this, we applied a novel approach using supervised machine learning to identify risk factors for DBS explant within the first two years following placement.

## 2. Materials and Methods

The study was designed in accordance with TRIPOD-AI guidelines for reporting clinical prediction models that use regression or machine learning methods.

### 2.1. Data

Data were obtained via a Data Use Agreement between the authors and the University of California, Irvine Medical Center. Data were collected from the Medical Informatics Operating Room Vitals and Events Repository (MOVER) database [15], which contains de-identified electronic health record data from 58,799 unique patients who underwent surgery at the University of California, Irvine Medical Center. Data within the MOVER database are de-identified in accordance with the HIPAA Privacy Rule; therefore, patient consent was not obtained.

### 2.2. Participants

**Figure 1:**
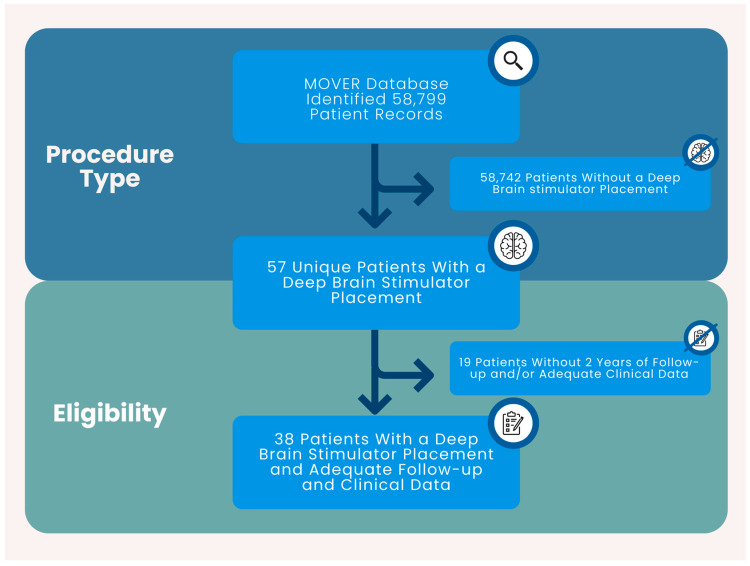
The cohort selection process yielded 38 patients with a DBS procedure and adequate clinical data.

Records included in this study involved patients with a DBS stimulator placement at a single academic center. Treatment consisted of DBS placement. Inclusion criteria: adult (≥18 years of age), DBS procedure, and at least two years of follow-up. Exclusion Criteria: <18 years of age, <2 years of follow-up, and no DBS procedure. In total, 38 unique patients were included in this study (Figure 1).

### 2.3. Data Preparation

Variables were created using International Classification of Diseases (ICD)-10-CM codes, patient demographics, the American Society of Anesthesiologists (ASA) score, anesthesia type, and postoperative events, including hospitalizations and intensive care unit (ICU) admissions. The included codes and/or definitions for each of the study variables can be seen in Table S1. Sex was encoded: 1: female and 0: male. Medical comorbidities were encoded/; 1: present and 0: absent. Anesthesia type was encoded: 1: monitored anesthesia care and 0: general anesthesia. ICU admission was encoded: 1: yes and 0: no. LOS and the ASA score was encoded as the numerical value.

### 2.4. Predictors

Predictors were chosen broadly, as the methodology using recursive factor elimination with cross-validation would remove variables offering limited predictive value based on statistical contribution. The surgical approach was defined by the method of lead insertion as either percutaneous (ICD 00H00MZ) or open (00H03MZ).

### 2.5. Sample Size

All patients meeting inclusion/exclusion criteria were included. The study cohort consisted of 38 patient records.

### 2.6. Missing Data

No records had missing data.

### 2.7. Analytical Methods

**Figure 2:**
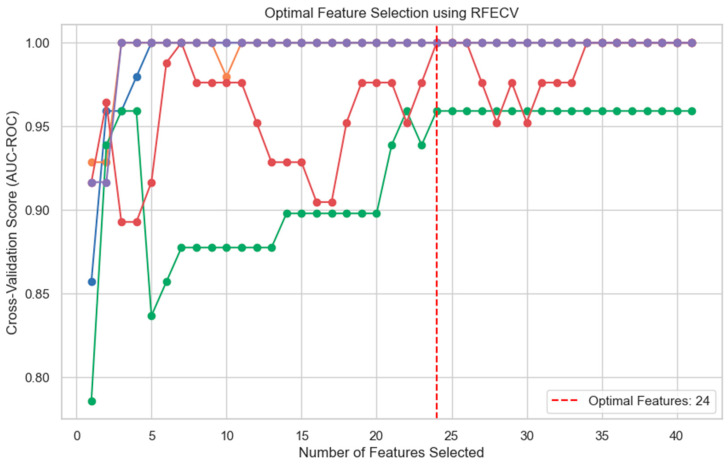
Five unique iterations of recursive factor elimination were performed, with the model identifying 24 factors as valuable predictors based upon the resultant AUC. Each individual-colored line represents a single iteration of recursive factor elimination. This approach minimizes the risk of overfitting and ensures only factors offering substantial predictive value are included in the model.

The data analysis plan was written prior to accessing the data. Continuous variables were reported as means ± standard deviation (SD) or medians ± interquartile range (IQR). Categorical variables were reported as *n*-values and percentages. Initial analysis consisted of comparing patients with DBS explantation to those without explantation using Fisher’s exact test for categorical variables and the Mann–Whitney U test for continuous variables. Statistical significance was set at *p* < 0.05. Next, the data were imported into Anaconda Version 2.3.1. (Anaconda Software Distribution. Austin, TX, USA). The following add-ons were used for analysis: pandas [16], numpy, sklearn [17], scikit-learn-extra [17], matplotlib. pyplot [18], and seaborn [19]. The target variable (explantation or no explantation) was defined, and the features were scaled. The Synthetic Minority Oversampling Technique (SMOTE) was used to generate synthetic examples of the minority class by interpolating between existing minority class samples and their nearest neighbors in feature space. The SMOTE-balanced data were then used to train a logistic regression model with L2 regularization. To define the optimal features to include in the model, recursive feature elimination with cross-validation (RFECV) was imported from sklearn. feature_selection, and the cross_val_score was imported from sklearn. model_selection [17]. Five iterations of RFECV with different data splits were performed, and the ideal number of features (24) was defined using the optimal average area under the receiver operating curve (AUC) (Figure 2). The data were then limited to the 24 selected features, and random_state was used to split the data into training (80%) and testing (20%) using deterministic train–test sets. The test data were not used in the training of the model. The logistic model was fitted, and performance was assessed using precision, recall, F1-score, and AUC. The model was then used to define odds ratios and 95% confidence intervals for each of the variables. The variables were considered to convey a statistically significant increased risk if the odds ratio and the entire 95% confidence interval were greater than 1.0.

### 2.8. Class Imbalance

Class imbalance was mitigated using SMOTE.

### 2.9. Fairness

Five iterations of recursive factor elimination with cross-validation were applied to minimize the inherent risk of overfitting.

### 2.10. Model Output

The output consisted of an odds ratio and a 95% confidence interval. In rare outcomes, the odds ratio approximates relative risk, making this an appropriate measure for the study. Statistical significance was based upon an odds ratio and 95% confidence interval >1.0 or <1.0.

## 3. Results

### 3.1. Cohort Demographics

Baseline cohort characteristics can be seen in Table 1. The study cohort was 65.8% male with an average age of 64.8 (+/− 11.6) years. In total, 5 of the 38 included patients (13%) had DBS explantation. The most common indication was primary PD (78.9%), followed by dystonia (23.7%). Almost all cases were performed under general anesthesia (97.4%), with patients having a median ASA score of 3.0 (IQR: 3.0–3.0). The median length of stay was 1.0 (IQR 1.0–1.3) days, with most patients requiring ICU admission (94.7%). No statistically significant differences were observed in demographic or perioperative variables. The most common medical comorbidities seen were hyperlipidemia (18.4%), sleep disorders (15.8%), dysautonomia (15.8%), obesity (15.8%), and a personal history of malignancy (15.8%). Chronic pain was more frequently seen in patients with DBS explantation than those without (*p* = 0.0108); however, no other statistically significant differences were noted. Amongst psychiatric and social comorbidities, tobacco use (31.2%), anxiety (21.1%), major depressive disorder (15.8%), and opioid use (5.3%) were present. However, only tobacco use was significantly higher in patients with explants (*p* = 0.0026). No other statistically significant differences were seen between the cohorts. The reason for explantation was infection and/or inflammation in two patients, surgical wound disruption in one patient, and was unavailable for two patients.

### 3.2. Multivariate Logistic Regression Model

As patients’ variables do not exist in isolation, we hypothesized a model that could account for the interaction between variables and may more accurately uncover potential perioperative risk factors. To test this hypothesis, we applied supervised machine learning using RFECV and a multivariate logistic regression model. The logistic regression model displayed robust performance with an average precision of 0.89, an average recall of 0.86, an average F1-score of 0.86, and an AUC-ROC of 1.0.

**Figure 3:**
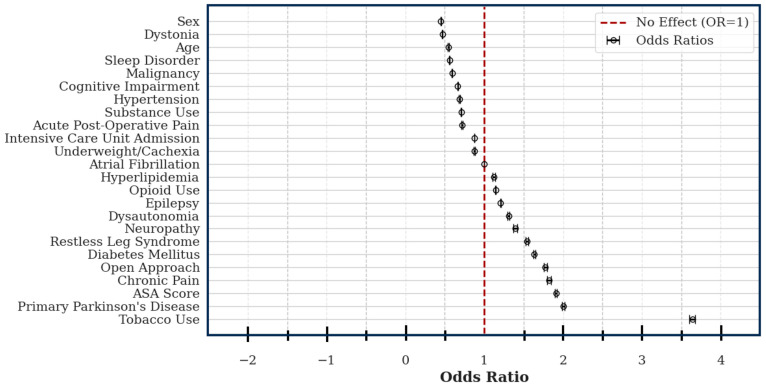
Forest plot illustrating the odds ratio (circle) and 95% confidence (error bars) interval for each of the included variables. Hyperlipidemia, opioid use, epilepsy, dysautonomia, neuropathy, restless leg syndrome, diabetes, an open surgical approach, chronic pain, an increased ASA score, an indication of primary PD, and tobacco use all conveyed increased risk of explant.

Amongst the assessed variables, 12 conveyed an increased risk of explant: tobacco use (OR: 3.64; CI: 3.60–3.68), primary PD (OR: 2.01; CI: 1.99–2.02), ASA score (OR: 1.91; CI: 1.90–1.92), chronic pain (OR: 1.82; CI: 1.80–1.85), diabetes (OR: 1.63; CI: 1.62–1.65), restless leg syndrome (OR: 1.55; CI: 1.53–1.56), neuropathy (OR: 1.40; CI: 1.37–1.42), dysautonomia (OR: 1.31; CI: 1.30–1.32), epilepsy (OR: 1.21; CI: 1.20–1.21), a history of opioid use (OR: 1.14; CI: 1.14–1.15), and hyperlipidemia (OR: 1.12; CI: 1.11–1.14). Female sex (OR: 0.45; CI: 0.45–0.45), an indication of dystonia (OR: 0.47; CI: 0.46–0.47), patient age (OR: 0.55; CI: 0.54–0.55), sleep disorders (OR: 0.56; CI: 0.55–0.56), history of malignancy (OR: 0.59; CI: 0.59–0.60), cognitive impairment (OR: 0.66; CI; 0.66–0.67), hypertension (OR: 0.69; CI: 0.68–0.69), illicit substance use (OR: 0.71; CI: 0.70–0.71), acute postoperative pain (OR: 0.72; CI: 0.71–0.72), ICU admission (OR: 0.87; CI: 0.87–0.88), comorbid cachexia/underweight (OR: 0.88; CI: 0.87–0.88), and atrial fibrillation (OR: 1.00; CI: 1.00–1.00) were included in the model; however, they all conveyed decreased or unchanged risk (Figure 3).

## 4. Discussion

Since its advent as a viable treatment for PD, deep brain stimulation has continued to grow in indications and number of procedures [3,4,5,6]. While significant advances have been made in increasing the precision of DBS technology itself, rates of hardware-related complications [20] and explantation [21] remain unacceptably high, suggesting a need to more precisely identify patients at risk of adverse outcomes [22]. Here, we sought to address this challenge by taking a unique approach using supervised machine learning in the form of a multivariate logistic regression combined with recursive factor elimination with cross-validation. In doing so, we identified a history of tobacco use, an indication of primary PD, an increased ASA score, comorbid chronic pain, an open surgical approach, comorbid diabetes, comorbid restless leg syndrome, comorbid neuropathy, dysautonomia, comorbid epilepsy, a history of opioid use, and comorbid hyperlipidemia as potential risk factors for early DBS explantation. While the purpose of this study was not to identify generalizable risk factors but rather to illustrate a novel methodology, the observed increase in explant rates among patients with tobacco use and comorbid diabetes may be attributable to a higher risk of postoperative infection. Specifically within DBS surgery, both a history of preoperative smoking and diabetes have been associated with an increased infection risk [23,24,25].

A national study conducted in Korea identified older age, male sex, comorbid dementia, and comorbid fractures as factors predictive of morbidity following DBS placement [14]. Similarly, Ward et al. identified ineffective stimulation (28%), lead dysfunction (30%), and infections (28%) as etiologies of DBS failure [10]. A retrospective, single-institution study using basic statistical analysis identified the use of topical vancomycin (as opposed to intrawound) as a risk factor for DBS infection [20]. A second single-center retrospective study identified the use of general anesthesia, hypertension, heart disease, and depression as risk factors for increased postoperative length of stay [26]. Furthermore, while associations with explant were limited, they did note an association with preoperative body mass index and risk of explant [26]. A retrospective study by Deeb et al. assessing the association between psychiatric comorbidities and DBS explantation in a Tourette syndrome database found no statistically significant associations [21].

Compared to previous studies, our study included a broader range of risk factors. The rationale for this was two-fold. First, given that the statistical plan involved the application of recursive factor elimination to regress out non-predictive variables, we chose to err on the side of a broad number of factors. Second, we wished to consider factors that had not previously been studied. For example, drawing from previous work by the authors [27] and others [28], we suspected chronic pain could have a deleterious effect on non-pain-directed neuromodulation.

Limitations of our study include the limited number of patients, the retrospective design, and the use of ICD codes, which limits the ability to capture granular clinical reasoning [29] and may be subject to inaccuracies, and the inclusion of a singular center. Furthermore, the rate of explant within the MOVER database was higher (13%) than values typically reported in the literature [21], suggesting that single-center limitations may have influenced this finding. Finally, given the small sample size and narrow confidence intervals (tobacco use CI: 3.60–3.68, AUC: 1.0), it is likely that there was some degree of overfitting. While many steps were taken to minimize this risk (RFECV), it should be considered in the interpretation of the results. The strengths of the study include the robust number of assessed variables (40), the use of a supervised machine learning model allowing for the assessment of inter-variables interactions, and the use of recursive factor elimination with cross-validation to identify the optimal variables. Importantly, this study offers a framework that could allow for the creation of a data-driven perioperative screen protocol, allowing for the identification and optimization of risk factors prior to surgery. In addition, this study helps us identify a subset of the patient population that may be at a higher risk for explant. We would recommend the preoperative optimization of risk factors like tobacco use and the optimization of blood sugar levels, hyperlipidemia, and opiate use before DBS surgery for better results.

## 5. Conclusions

The identification of risk factors for DBS explant has proven challenging using basic statistical models, which are not well adapted for rare outcomes. Here, we offer a novel methodology using an optimized multivariable logistic regression, which may offer a solution to this challenge. While we chose to share results for the purpose of validating the functionality of the model, further studies, with a larger number of patients, are necessary to validate the clinical utility of these associations.

## Figures and Tables

**Table 1:** Cohort demographics and clinical data.

	DBS	Explant	No Explant	*p*-Value
Number of Patients	38	5 (13.2%)	33 (86.8%)	0.1440
Sex				0.1440
Male *n* (%)	25 (65.8%)	5 (100.0%)	20 (60.6%)	0.4416
Female *n* (%)	13 (34.2%)	0 (0.0%)	13 (39.4%)	
Age (years +/− SD)	64.8 +/− 11.6	62.4 +/− 6.2	65.2 +/− 12.2	0.1316
Anesthesia Type				0.1316
General Anesthesia *n* (%)	37 (97.4%)	4 (80.0%)	33 (100.0%)	>0.9999
Monitored Airway *n* (%)	1 (2.6%)	1 (20.0%)	0 (0.0%)	0.8107
ASA Score (median, IQR)	3.0 (3.0–3.0)	3.0 (3.0–3.0)	3.0 (3.0–3.0)	0.2489
Length of Stay (median, IQR)	1.0 (1.0–1.3)	1.0 (1.0–1.5)	1.0 (1.0–1.5)	
ICU Admission *n* (%)	36 (94.73%)	4 (80.0%)	32 (97.0%)	>0.9999
Indication for DBS **				>0.9999
Primary PD *n* (%)	30 (78.94%)	4 (80.0%)	26 (78.8%)	0.1195
Secondary PD *n* (%)	1 (2.63%)	0 (0.0%)	1 (3.0%)	0.3121
Essential Tremor *n* (%)	5 (13.15%)	2 (40.0%)	3 (9.1%)	>0.9999
Dystonia *n* (%)	9 (23.68%)	0 (0.0%)	9 (27.3%)	
Spasticity *n* (%)	1(2.63%)	0 (0.0%)	1 (3.0%)	0.2440
Surgical Approach				0.2440
Percutaneous *n* (%)	29 (76.31%)	3 (60.0%)	26 (83.9%)	
Open *n* (%)	7 (18.42%)	2 (40.0%)	5 (16.1%)	0.1316
Medical Comorbidities				0.4456
Epilepsy (%)	1 (2.63%)	1 (20.0%)	0 (0.0%)	>0.9999
Neuropathy (%)	4 (10.52%)	1 (20.0%)	3 (9.1%)	0.0108 *
Acute Postoperative Pain (%)	2 (5.26%)	0 (0.0%)	2 (6.1%)	0.1690
Chronic Pain (%)	5 (13.15%)	3 (60.0%)	2 (6.1%)	
Dysautonomia (%)	6 (15.78%)	2 (40.0%)	4 (12.1%)	>0.9999
Chronic Fatigue (%)	0.0%	0.0%	0.0%	0.1316
Cognitive Impairment (%)	5 (13.15%)	0 (0.0%)	5 (15.2%)	>0.9999
Restless Leg Syndrome (%)	1 (2.63%)	1 (20.0%)	0 (0.0%)	0.1195
Cerebrovascular Disease (%)	1 (2.63%)	0 (0.0%)	1 (3.0%)	0.5701
Sleep Apnea (%)	5 (13.15%)	2 (40.0%)	3 (9.1%)	0.4456
Sleep Disorder, Any (%)	6 (15.78%)	0 (0.0%)	6 (18.2%)	>0.9999
Chronic Obstructive Pulmonary Disease (%)	4 (10.52%)	1 (20.0%)	3 (9.1%)	0.2227
Hypertension (%)	17 (44.73%)	2 (40.0%)	15 (45.5%)	>0.9999
Hyperlipidemia (%)	7 (18.42%)	2 (40.0%)	5 (15.2%)	0.1195
Atrial Fibrillation (%)	2 (5.26%)	0 (0.0%)	2 (6.1%)	0.5272
Diabetes Mellitus (%)	5 (13.15%)	2 (40.0%)	3 (9.1%)	0.2489
Chronic Kidney Disease (%)	5 (13.15%)	1 (20.0%)	4 (12.1%)	>0.9999
Fibromyalgia (%)	2 (5.26%)	1 (20.0%)	1 (3.0%)	>0.9999
Irritable Bowel Syndrome (%)	1 (2.63%)	0 (0.0%)	1 (3.0%)	0.1690
Underweight/Cachexia (%)	4 (10.52%)	0 (0.0%)	4 (12.1%)	>0.9999
Obesity (%)	6 (15.78%)	2 (40.0%)	4 (12.1%)	>0.9999
Migraine (%)	1 (2.63%)	0 (0.0%)	1 (3.0%)	0.5701
Urinary Incontinence (%)	3 (7.89%)	0 (0.0%)	3 (9.1%)	
Malignancy (%)	6 (15.78%)	0 (0.0%)	6 (18.2%)	
Bowel Incontinence (%)	0 (0.0%)	0 (0.0%)	0 (0.0%)	0.2489
Substance Use				>0.9999
Opioid Use (%)	2 (5.26%)	1 (20.0%)	1 (3.0%)	0.0026 *
Substance Use (%)	1 (2.63%)	0 (0.0%)	1 (3.0%)	
Tobacco Use (%)	13 (34.21%)	5 (100.0%)	8 (24.2%)	>0.9999
Psychiatric Comorbidities				>0.9999
Anxiety (%)	8 (21.05%)	1 (20.0%)	7 (21.2%)	
MDD (%)	6 (15.78%)	1 (20.0%)	5 (15.2%)	
ADHD (%)	0 (0.0%)	0 (0.0%)	0 (0.0%)	
ETOH (%)	0 (0.0%)	0 (0.0%)	0 (0.0%)	
OCD (%)	0 (0.0%)	0 (0.0%)	0 (0.0%)	
PTSD (%)	0 (0.0%)	0 (0.0%)	0 (0.0%)	

* = *p* < 0.05; ** = In patients with multiple listed indications, they were included for each of the indications; MDD = major depressive disorder; ADHD = Attention-deficit/hyperactivity disorder; ETOH = alcohol use disorder; OCD = Obsessive-Compulsive Disorder; PTSD = Post-traumatic stress disorder.

## Data Availability

The data that support the findings of this study are openly available in the MOVER database at https://mover.ics.uci.edu/index.html (accessed on 15 July 2025), reference [15].

## Supplementary Materials

The following supporting information can be downloaded at: https://www.mdpi.com/article/10.3390/clinpract15070132/s1, Table S1: Detailed definitions of all study variables.

## Abbreviations

The following abbreviations are used in this manuscript:

PD	Parkinson’s Disease
DBS	Deep brain stimulation
AUC	Area under the curve
RFECV	Recursive factor elimination with cross-validation
MOVER	Medical Informatics Operating Room Vitals and Events Repository
OR	Odds ratio
CI	95% confidence interval
ASA	American Society of Anesthesiologists
BMI	Body mass index
ICD	International Classification of Diseases
SMOTE	Synthetic Minority Oversampling Technique
SD	Standard deviation
IQR	Interquartile range
ICU	Intensive care unit
LOS	Length of stay

## References

[1] Benabid A.L., Pollak P., Louveau A., Henry S., de Rougemont J. (1987). Combined (thalamotomy and stimulation) stereotactic surgery of the VIM thalamic nucleus for bilateral Parkinson disease. Appl. Neurophysiol..

[2] Servello D., Galbiati T.F., Iess G., Minafra B., Porta M., Pacchetti C. (2023). Complications of deep brain stimulation in Parkinson’s disease: A single-center experience of 517 consecutive cases. Acta Neurochir..

[3] Hacker M.L., Turchan M., Heusinkveld L.E., Currie A.D., Millan S.H., Molinari A.L., Konrad P.E., Davis T.L., Phibbs F.T., Hedera P. (2020). Deep brain stimulation in early-stage Parkinson disease: Five-year outcomes. Neurology.

[4] Figee M., Riva-Posse P., Choi K.S., Bederson L., Mayberg H.S., Kopell B.H. (2022). Deep Brain Stimulation for Depression. Neurotherapeutics.

[5] Knotkova H., Hamani C., Sivanesan E., Le Beuffe M.F.E., Moon J.Y., Cohen S.P., Huntoon M.A. (2021). Neuromodulation for chronic pain. Lancet.

[6] Wang S., Fan S., Gan Y., Zhang Y., Gao Y., Xue T., Xie H., Ma R., Zhang Q., Zhao B. (2024). Efficacy and safety of combined deep brain stimulation with capsulotomy for comorbid motor and psychiatric symptoms in Tourette’s syndrome: Experience and evidence. Asian J. Psychiatr..

[7] Krauss J.K., Lipsman N., Aziz T., Boutet A., Brown P., Chang J.W., Davidson B., Grill W.M., Hariz M.I., Horn A. (2021). Technology of deep brain stimulation: Current status and future directions. Nat. Rev. Neurol..

[8] Deng H., Yue J.K., Wang D.D. (2021). Trends in safety and cost of deep brain stimulation for treatment of movement disorders in the United States: 2002–2014. Br. J. Neurosurg..

[9] Reich M.M., Hsu J., Ferguson M., Schaper F., Joutsa J., Roothans J., Nickl R.C., Frankemolle-Gilbert A., Alberts J., Volkmann J. (2022). A brain network for deep brain stimulation induced cognitive decline in Parkinson’s disease. Brain.

[10] Ward M., Ahmed M., Markosian C., Ezike J.Z., Agrawal R., Randhawa K., Liang Z., Abraham M., Paskhover B., Mammis A. (2021). Complications associated with deep brain stimulation for Parkinson’s disease: A MAUDE study. Br. J. Neurosurg..

[11] Olson M.C., Shill H., Ponce F., Aslam S. (2023). Deep brain stimulation in PD: Risk of complications, morbidity, and hospitalizations: A systematic review. Front. Aging Neurosci..

[12] Chen T., Mirzadeh Z., Lambert M., Gonzalez O., Moran A., Shetter A.G., Ponce F.A. (2017). Cost of Deep Brain Stimulation Infection Resulting in Explantation. Stereotact. Funct. Neurosurg..

[13] Jung I.H., Chang K.W., Park S.H., Chang W.S., Jung H.H., Chang J.W. (2022). Complications After Deep Brain Stimulation: A 21-Year Experience in 426 Patients. Front. Aging Neurosci..

[14] Kim A., Yang H.J., Kwon J.H., Kim M.H., Lee J., Jeon B. (2023). Mortality of Deep Brain Stimulation and Risk Factors in Patients With Parkinson’s Disease: A National Cohort Study in Korea. J. Korean Med. Sci..

[15] Samad M., Angel M., Rinehart J., Kanomata Y., Baldi P., Cannesson M. (2023). Medical Informatics Operating Room Vitals and Events Repository (MOVER): A public-access operating room database. JAMIA Open.

[16] McKinney W. Data Structures for Statistical Computing in Python. Proceedings of the 9th Python in Science Conference.

[17] Pedregosa F., Varoquaux G., Gramfort A., Michel V., Thirion B., Grisel O., Blondel M., Prettenhofer P., Weiss R., Dubourg V. (2011). Scikit-learn: Machine Learning in Python. J. Mach. Learn. Res..

[18] Hunter J.D. (2007). Matplotlib: A 2D graphics environment. Comput. Sci. Eng..

[19] Waskom M.L. (2021). seaborn: Statistical data visualization. J. Open Source Softw..

[20] Abode-Iyamah K.O., Chiang H.Y., Woodroffe R.W., Park B., Jareczek F.J., Nagahama Y., Winslow N., Herwaldt L.A., Greenlee J.D.W. (2019). Deep brain stimulation hardware-related infections: 10-year experience at a single institution. J. Neurosurg..

[21] Deeb W., Leentjens A.F.G., Mogilner A.Y., Servello D., Meng F., Zhang J., Galbiati T.F., Okun M.S. (2020). Deep brain stimulation lead removal in Tourette syndrome. Park. Relat. Disord..

[22] Scelzo E., Beghi E., Rosa M., Angrisano S., Antonini A., Bagella C., Bianchi E., Caputo E., Lena F., Lopiano L. (2019). Deep brain stimulation in Parkinson’s disease: A multicentric, long-term, observational pilot study. J. Neurol. Sci..

[23] Farrokhi F.R., Marsans M.T., Sikora M., Monsell S.E., Wright A.K., Palmer M., Hoefer A., McLeod P., Mark J., Carlson J. (2019). Pre-operative smoking history increases risk of infection in deep brain stimulation surgery. J. Clin. Neurosci..

[24] Bernstein J.E., Kashyap S., Ray K., Ananda A. (2019). Infections in Deep Brain Stimulator Surgery. Cureus.

[25] Bjerknes S., Skogseid I.M., Sæhle T., Dietrichs E., Toft M. (2014). Surgical site infections after deep brain stimulation surgery: Frequency, characteristics and management in a 10-year period. PLoS ONE.

[26] Tiefenbach J., Kuvliev E., Dullur P., Mandava N., Hogue O., Kondylis E., Sharma A., Rammo R., Nagel S., Machado A.G. (2024). The Rate and Risk Factors of Deep Brain Stimulation-Associated Complications: A Single-Center Experience. Oper. Neurosurg..

[27] Murin P.J., Murin P.J., Lima de Mendonca Y., Martins Y.C. (2025). Identification of Perioperative Risk Factors for Early Sacral Nerve Stimulator Explantation: A Single-Center Retrospective Cohort Study. J. Clin. Med..

[28] Shaheen N., Shaheen A., Elgendy A., Bezchlibnyk Y.B., Zesiewicz T., Dalm B., Jain J., Green A.L., Aziz T.Z., Flouty O. (2023). Deep brain stimulation for chronic pain: A systematic review and meta-analysis. Front. Hum. Neurosci..

[29] Hussain N., Weaver T. (2023). Response to the Letter to the Editor Regarding: “Identifying Predictors for Early Percutaneous Spinal Cord Stimulator Explant at One and Two Years: A Retrospective Database Analysis”. Neuromodulation.

