# Supplementary material for: Optimizing Multivariable Logistic Regression for Identifying Perioperative Risk Factors for Deep Brain Stimulator Explantation: A Pilot Study
Source: Clin Pract. 2025 Jul 17;15(7):132. doi: 10.3390/clinpract15070132 (PMC12293133; doi:10.3390/clinpract15070132)
Supplement: Supplementary file 1 [file clinpract-15-00132-s001.zip › clinpract-3613999-supplementary.pdf]

Table S1: Data Collection Sheet

Variable	Definition
Age at time of implant	Age in years
Gender	Female: 1, Male: 0
Deep Brain Stimulator Insertion	CPT: 61885, 61886<br>Percutaneous Approach: ICD 00H03MZ<br>Open Approach: ICD 00H00MZ
Deep Brain Stimulator Removal	ICD-10: 00P03MZ, 00P00MZ
ASA score	American Society of Anaesthesiologist Score (1-5)
Length of Stay	Length of stay in days following implantation for any reason.
ICU Admission	Admission to the ICU following implantation for any reason.
Deep Brain Stimulator Indication	Primary Parkinson's Disease (ICD G20), Secondary Parkinson's Disease (ICD G21.9), Essential Tremor (ICD G25.0), Dystonia (ICD G24.9), Spasticity (ICD G11.4)
Past Medical History	Medical comorbidity data will be queried using ICD-10 codes, with assessed diagnoses including epilepsy (ICD G40.89), neuropathy (ICD G62.9, M54.16, G50.0, E11.42), acute post-operative pain (ICD G89.18), chronic pain (ICD G90.511, G89.29, M54.5), dysautonomia (ICD I95.1, G90.8, H04.123, K59.09,G90.511, K31.84), chronic fatigue (ICD R53.82), cognitive impairment (ICD G31.84, F02.80, R40.3), restless leg syndrome (ICD G25.81), cerebrovascular disease (ICD Z86.73), sleep apnoea (ICD G47.30, G47.33, G47.39), sleep disorder (ICD G47.00, G47.30, G47.33, G47.39), chronic obstructive pulmonary disease (ICD J44.9), hypertension (ICD I10), hyperlipidaemia (ICD E78.5, E78.1, E78.00), atrial fibrillation (ICD I48.91), diabetes mellitus (ICD E11.9, E11.42, E11.21), chronic kidney disease (ICD I12.9, N18.9, N18.2, N18.3), anxiety (ICD F41.9, F41.8), depression (ICD F32.9, F32.89), attention deficit hyperactivity disorder (ICD F90.9), fibromyalgia (ICD M79.7), irritable bowel syndrome (ICD K58.9), underweight (ICD R64, R63.6), obesity (ICD E66.01, E66.9, Z68.35, Z68.36, E66.8), migraine (ICD G43.909), urinary incontinence (ICD R32, R33.8, R33.9), bowel incontinence (ICD R15.9), opioid use (ICD F11.90, Z79.891), substance use (ICD F12.90), alcohol use

	(ICD F10.9), tobacco use (ICD Z87.891, F17.200), obsessive compulsive disorder (ICD F42), post-traumatic stress disorder (ICD F43.1), malignancy (ICD Z85.46, Z85.038, Z85.3, Z85.89, Z85.828)
Reason for Deep Brain Stimulator Explantation	Infection/Inflammatory Reaction: ICD T85.731A<br>Surgical Wound Disruption: ICD T81.31XA
